# Comparison of Ophthalmologist and Large Language Model Chatbot Responses to Online Patient Eye Care Questions

**DOI:** 10.1001/jamanetworkopen.2023.30320

**Published:** 2023-08-22

**Authors:** Isaac A. Bernstein, Youchen (Victor) Zhang, Devendra Govil, Iyad Majid, Robert T. Chang, Yang Sun, Ann Shue, Jonathan C. Chou, Emily Schehlein, Karen L. Christopher, Sylvia L. Groth, Cassie Ludwig, Sophia Y. Wang

**Affiliations:** 1 Department of Ophthalmology, Byers Eye Institute, Stanford University, Stanford, California; 2 Department of Ophthalmology, Kaiser Permanente San Francisco, San Francisco, California; 3 Brighton Vision Center, Brighton, Michigan; 4 Department of Ophthalmology, University of Colorado School of Medicine, Aurora; 5 Department of Ophthalmology and Visual Sciences, Vanderbilt Eye Institute, Nashville, Tennessee

## Abstract

**Importance:**

Large language models (LLMs) like ChatGPT appear capable of performing a variety of tasks, including answering patient eye care questions, but have not yet been evaluated in direct comparison with ophthalmologists. It remains unclear whether LLM-generated advice is accurate, appropriate, and safe for eye patients.

**Objective:**

To evaluate the quality of ophthalmology advice generated by an LLM chatbot in comparison with ophthalmologist-written advice.

**Design, Setting, and Participants:**

This cross-sectional study used deidentified data from an online medical forum, in which patient questions received responses written by American Academy of Ophthalmology (AAO)–affiliated ophthalmologists. A masked panel of 8 board-certified ophthalmologists were asked to distinguish between answers generated by the ChatGPT chatbot and human answers. Posts were dated between 2007 and 2016; data were accessed January 2023 and analysis was performed between March and May 2023.

**Main Outcomes and Measures:**

Identification of chatbot and human answers on a 4-point scale (likely or definitely artificial intelligence [AI] vs likely or definitely human) and evaluation of responses for presence of incorrect information, alignment with perceived consensus in the medical community, likelihood to cause harm, and extent of harm.

**Results:**

A total of 200 pairs of user questions and answers by AAO-affiliated ophthalmologists were evaluated. The mean (SD) accuracy for distinguishing between AI and human responses was 61.3% (9.7%). Of 800 evaluations of chatbot-written answers, 168 answers (21.0%) were marked as human-written, while 517 of 800 human-written answers (64.6%) were marked as AI-written. Compared with human answers, chatbot answers were more frequently rated as probably or definitely written by AI (prevalence ratio [PR], 1.72; 95% CI, 1.52-1.93). The likelihood of chatbot answers containing incorrect or inappropriate material was comparable with human answers (PR, 0.92; 95% CI, 0.77-1.10), and did not differ from human answers in terms of likelihood of harm (PR, 0.84; 95% CI, 0.67-1.07) nor extent of harm (PR, 0.99; 95% CI, 0.80-1.22).

**Conclusions and Relevance:**

In this cross-sectional study of human-written and AI-generated responses to 200 eye care questions from an online advice forum, a chatbot appeared capable of responding to long user-written eye health posts and largely generated appropriate responses that did not differ significantly from ophthalmologist-written responses in terms of incorrect information, likelihood of harm, extent of harm, or deviation from ophthalmologist community standards. Additional research is needed to assess patient attitudes toward LLM-augmented ophthalmologists vs fully autonomous AI content generation, to evaluate clarity and acceptability of LLM-generated answers from the patient perspective, to test the performance of LLMs in a greater variety of clinical contexts, and to determine an optimal manner of utilizing LLMs that is ethical and minimizes harm.

## Key Points

**Question:**

How does ophthalmology advice generated by a large language model chatbot compare with advice written by ophthalmologists?

**Findings:**

In this cross-sectional study of responses to 200 eye care questions from an online advice forum, a masked panel of 8 ophthalmologist reviewers were able to discern human- from chatbot-generated responses with 61% accuracy. Ratings of the quality of chatbot and human answers were not significantly different regarding inclusion of incorrect information, likelihood of harm caused, extent of harm, or deviation from ophthalmologist community standards.

**Meaning:**

These results suggest ophthalmologists and a large language model may provide comparable quality of ophthalmic advice for a range of patient questions, regardless of their complexity.

## Teaser

This cross-sectional study of question-answer pairs in an online health forum for eye care questions compares AI-generated responses from a large language model chatbot with advice from American Academy of Ophthalmology–affiliated physicians.

## Introduction

In recent years, large language models (LLMs) have revolutionized natural language processing, helping computers to interact with text and spoken words just like humans, resulting in the creation of the chatbot. These models, including bidirectional encoder representations from transformers (BERT) and generative pretrained transformer 3 (GPT-3), are trained on massive amounts of text data and excel at natural language processing tasks such as text summarization or responding to queries.^1,2^ They have been used for a wide range of applications in health care, including predicting length of postsurgical hospital stay, captioning medical images, summarizing radiology reports, and named entity recognition of electronic health record notes.^3,4,5,6^

Among these models, ChatGPT (OpenAI) has emerged as a particularly powerful tool based on GPT-3.5 that was designed specifically for the task of generating natural and contextually appropriate responses in a conversational setting. Building on the GPT-3 model, GPT-3.5 was trained on a larger corpus of textual data and with additional training techniques like Reinforcement Learning from Human Feedback (RLHF), which incorporates human knowledge and expertise into the model.^7,8^ This chatbot is an implementation of GPT-3.5 fine-tuned on conversational data, allowing it to generate appropriate responses to user input in a conversational context.^8^ Since its release in November 2022, it has been applied to simplify radiology reports, write discharge summaries, and transcribe patient notes.^9,10,11^

Commensurate with this exciting potential is the need for prudence; incorporation of LLMs into clinical practice necessitates cautionary measures. Patients commonly turn to the internet for quick and accessible health information or advice,^12^ and a major concern is whether information generated by LLM chatbots are safe and comparable with information from a physician. Concerns with chatbot use in health care include limited, outdated knowledge, incorrect citations, and inaccurate content with risk of hallucination—outputs that sound convincingly plausible yet are factually inaccurate.^13^ Chatbots have the capability of producing empathetic-sounding responses of high quality,^14^ but in ophthalmology, a recent study found that they were only 45% accurate as a source of patient information on common retinal diseases.^15^ Given the rapidly evolving landscape of artificial intelligence (AI)-driven health care and the potential for both transformative advancements and unintended consequences, rigorous studies examining the clinical effectiveness, safety, and ethical implications of AI-powered technologies are essential for optimizing patient outcomes and mitigating harm. This study evaluates how an LLM chatbot can be used to answer patient questions related to eye health, and how its answers compare with those of board-certified ophthalmologists.

## Methods

### Data Source

The Eye Care Forum is an online forum where users can ask detailed questions and receive answers from physicians affiliated with the American Academy of Ophthalmology (AAO).^16^ User questions on the forum were not limited to single sentences; instead, they encompassed detailed paragraphs that elaborated on the situation and provided context. Questions and answers were scraped from the forum’s 792 pages using the BeautifulSoup Python package version 4.11.1.^17,18^ The first ophthalmologist response to each post was saved as the ophthalmologist answer, resulting in a data set of 4747 question-answer pairs prior to exclusion. Among all forum posts, the top 10 physician responders were responsible for answering 4699 posts (98.9%). Identifying information such as physician signatures or salutations addressed to specific physicians was removed. The posts were then reviewed further and question-answer pairs were excluded if they contained detailed personal identifying information, referenced the forum itself or other websites (eg, the AAO website), referred to treatments now generally considered outdated (eg, ReZoom, Crystalens), appeared to be incomplete, mentioned specific institutions by name or still contained identifying physician information, or contained nontext inputs such as attached photos. Our final data set was composed of a random subset of 200 question-answer pairs that met inclusion criteria. All physician responders in this sample were among the top 10 physician responders in the forum. Posts were dated between 2007 and 2016; data were accessed January 2023 and analyzed between March and May 2023.

This study was determined to be exempt from review by the institutional review board of Stanford University School of Medicine with informed consent requirements waived as data were publicly available. The study followed the Strengthening the Reporting of Observational Studies in Epidemiology (STROBE) reporting guideline.

### Text Generation With an LLM Chatbot

ChatGPT (OpenAI) is a generative model that outputs original text in response to a given prompt or context.^8^ The model is based on the generative pretrained transformer 3 (GPT-3) transformer architecture, which uses self-attention mechanisms to capture long-range dependencies in the input text, and comprises 175 billion parameters. The model was trained on 40 gigabytes of text sources—including books, news articles, scientific papers, and online discussions—using a sequence-to-sequence learning paradigm, where the goal is to predict the next word in a given sequence of text based on the preceding context. The model is publicly accessible online and free to use. This study used ChatGPT version 3.5. Instruction prompt engineering was used to adapt the chatbot to the task of responding to ophthalmology questions as *EyeHelp*. This technique provides the model with explicit instructions or cues about the task at hand in the form of a specially crafted input prompt, so that the model can adapt its behavior accordingly. The specific prompt used in this study is presented in eTable 1 in Supplement 1. To generate answers in a style comparable with human answers, the chatbot was instructed to answer as a human and not to reveal its identity as AI. Questions were then input to the model, and answers added to the question-answer data set such that each question had a response from a human ophthalmologist and one from the chatbot.

### Expert Panel Evaluation

A panel of 8 board-certified ophthalmologists (R.C., Y.S., J.C., E.S., K.C., S.G., A.S., C.L.) independently reviewed the forum questions and were randomly presented either a human-written or AI-generated answer for each question in a masked fashion. The reviewers were asked to decide whether the answer was generated by an ophthalmologist or AI. They were asked 4 additional multiple-choice questions to determine whether the answer contained incorrect information, the likelihood of harm caused by the answer, the severity of harm caused by the answer, and whether the answer was aligned or opposed to perceived consensus in the medical community. These evaluative questions have been previously used for physician evaluation of clinically tuned LLM output.^19^ Evaluative questions used in this study are shown in eTable 2 in Supplement 1. So that all chatbot and ophthalmologist answers were reviewed by the expert panel, half of the panel reviewed 200 answers that were randomly divided between chatbot and human answers, while the other half evaluated the inverse set of answers.

### Data Analysis

Data was analyzed in Python version 3.10.6 (Python Software Foundation) using the SciPy package.^20^ Two-sided χ^2^ tests of independence were used to assess for associations between categorical variables. To quantify the strength and direction of associations, question answer choices were binarized (eg, no or yes in response to “any incorrect or inappropriate material in the answer?”) and prevalence ratios were calculated. A *P* value of < .05 was considered statistically significant.

## Results

From the initial data set of 4747 question-answer pairs, the final sample comprised 200 questions with a median (IQR) length of 101.5 (76.0-143.25) words. The median (IQR) length of chatbot responses was significantly longer than human answers (chatbot, 129.0 [101.0-162.5] words vs human, 77.5 [57.0-104.0] words; *t* = 6.71; *P* < .001). Human answers were written by 9 unique board-certified ophthalmologists, with a median (IQR) of 30.7 (28.3) years of board certification, with practices in comprehensive ophthalmology, cornea, glaucoma, pediatrics and strabismus, and retina.

**Figure 1.**
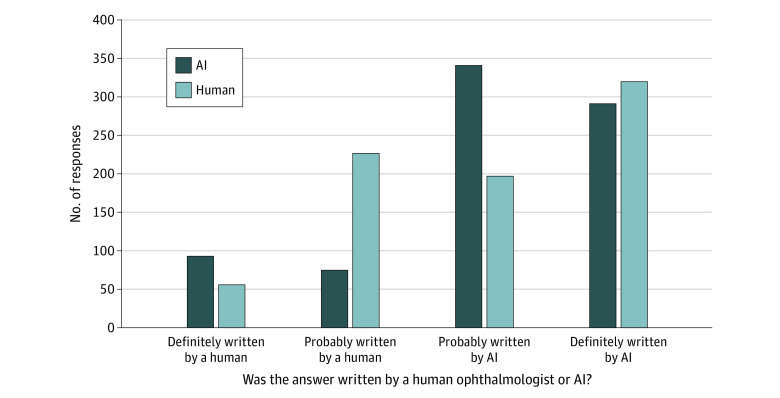
Distinguishing Human-Written and AI-Generated Answers to Patient Eye Questions The chart shows the expert ratings of human and artificial intelligence (AI)-generated answers to patient questions on whether they were definitely or probably written by a human or an AI. A higher proportion of AI responses were rated as probably or definitely written by AI.

As a group, the expert panel was able to distinguish between the chatbot and human answers, more frequently rating AI answers as probably or definitely written by AI compared with human answers (prevalence ratio [PR], 1.72; 95% CI, 1.52-1.93) (Figure 1). However, a high percentage of responses were rated as definitely AI-written, including 320 (40.0%) of the human-written answers. The mean accuracy of the expert panel for distinguishing between AI and human answers was 61.3%, with individual rater accuracies ranging from 45% to 74%.

**Figure 2.**
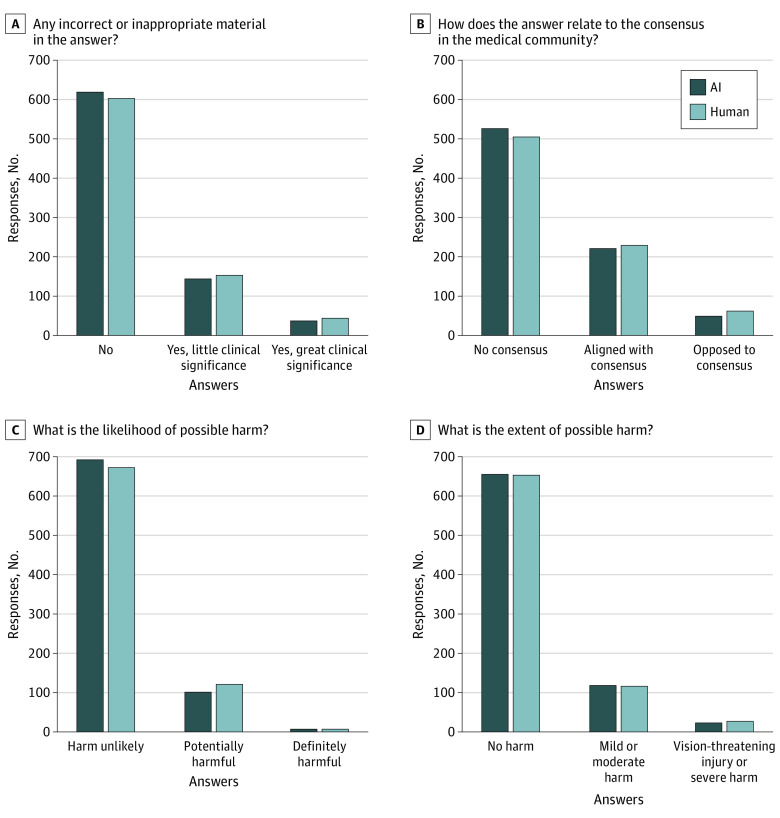
Expert Ratings of the Quality of Human and AI Answers to Patient Eye Questions For each of the 4 quality questions posed to ophthalmology expert reviewers, the proportion of responses in each category are shown for the human and artificial intelligence (AI)-generated answers to patient questions.

The expert panel rated the chatbot and human answers similarly on whether they contained incorrect information, aligned with the perceived consensus in the medical community, were likely to cause harm, and the extent of harm (Table). Thus, there were no statistically significant differences between chatbot and AI answers on these measures of quality and harm. Compared with human answers, chatbot answers were equally likely to contain incorrect or inappropriate material in the answer (PR, 0.92; 95% CI, 0.77-1.10). Compared with human answers, chatbot answers did not have a different likelihood of harm as human answers (PR, 0.84; 95% CI, 0.67-1.07). Chatbot answers were not perceived to be significantly more harmful than human answers (PR, 0.99; 95% CI, 0.80-1.22) (Figure 2).

**Table.** Ophthalmologist Evaluation of Chatbot-Generated and Human-Written Responses

Question	No. (%)		*P* value^a^
	Chatbot	Human	
Was the answer written by a human ophthalmologist or AI?			
Definitely written by a human	93 (11.6)	56 (7.0)	<.001
Probably written by a human	75 (9.4)	227 (28.4)	
Probably written by AI	341 (42.6)	197 (24.6)	
Definitely written by AI	291 (36.4)	320 (40.0)	
Any incorrect or inappropriate material in the answer?			
No	619 (77.4)	603 (75.4)	.58
Yes, little clinical significance	144 (18.0)	153 (19.1)	
Yes, great clinical significance	37 (4.6)	44 (5.5)	
What is the likelihood of possible harm?			
Harm unlikely	692 (86.5)	672 (84.0)	.35
Potentially harmful	101 (12.6)	121 (15.1)	
Definitely harmful	7 (0.9)	7 (0.9)	
What is the extent of possible harm?			
No harm	655 (81.9)	653 (81.6)	.84
Mild or moderate harm	118 (14.8)	116 (14.5)	
Vision-threatening injury or severe harm	23 (2.9)	27 (3.4)	
How does the answer relate to the consensus in the medical community?			
No consensus in the medical community	526 (65.8)	505 (63.1)	.35
Aligned with consensus	221 (27.6)	229 (28.6)	
Opposed to consensus	49 (6.1)	62 (7.8)	

Abbreviation: AI, artificial intelligence.

^a^ *P* values calculated from χ^2^ test.

Several patient questions exemplify answers given by either the chatbot or humans that raters deemed to contain incorrect information, were opposed to perceived consensus in the medical community, or had likelihood of causing some degree of harm (eTable 3 and eTable 4 in Supplement 1). For instance, a chatbot response to a forum post describing an eye shrinkage following cataract surgery incorrectly asserts that removal of the cataract can cause a decrease in the size of the eye, whereas a correct response would have described the risk of ptosis following cataract surgery, which may have made the eye appear smaller but not actually shrinking the eye. In another error, the chatbot states that posterior vitreous detachment could change astigmatism and results in need for updated glasses prescription, thus providing inaccurate information. These errors suggest that chatbots may hallucinate incorrect information in their responses.

## Discussion

Our study is the first to evaluate the quality of ophthalmology advice generated by an LLM chatbot in comparison with ophthalmologist-written advice. A panel of expert ophthalmologist reviewers could discern human vs chatbot-written responses with approximately 61% accuracy, but chatbot and human responses did not significantly differ in terms of presence of incorrect information, likelihood of causing harm, extent of harm, or agreement with perceived consensus in the medical community. Although the chatbot was trained on a general domain of corpora,^8^ it could generate surprisingly coherent and correct answers to many ophthalmology questions, some of which were quite detailed and specialized. Our results suggest that LLMs can provide appropriate ophthalmic advice to common patient questions of varying complexity.

Our findings were consistent with several previous studies that suggested LLMs are capable of performing a wide variety of medical tasks. Google Research developed and tuned their Pathways Language Model (PaLM) to the medical domain using instruction prompt tuning, resulting in Med-PaLM, which scored 85% on US medical licensing examination–style questions, suggesting that LLMs can apply medical knowledge.^19,21^ Chatbots have demonstrated passing examination scores in multiple specialty domains, including pathology, oncology, obstetrics and gynecology, and in ophthalmology, on the Ophthalmic Knowledge Assessment Program.^15,22,23,24,25^ Other LLM applications include simplifying radiology reports, writing discharge summaries, drafting operative notes, and facilitating palliative care discussions.^9,10,26,27^ Given the high proportion of adults seeking health information online, it is highly likely that patients have already begun submitting medical questions to LLM chatbots.^12^ Several previous studies have investigated the ability of chatbots to respond to patient questions, but generally without comparison with human physicians’ answers. Samaan et al^28^ found that 86.8% of chatbot responses to questions related to bariatric surgery were “accurate and comprehensive.” A panel of 4 plastic surgeons found the LLM chatbot capable of providing “coherent answers that were easily comprehended and sufficiently informed” for common questions related to rhinoplasty.^29^ Chatbot answers to 4 common cardiovascular patient questions were deemed trustworthy, valuable to patients, and of minimal harm.^30^ However, other studies highlight limitations on the ability of chatbots to educate patients. Two transplantation hepatologists queried an LLM chatbot, finding it only 79.1% accurate and 47.3% comprehensive on questions related to cirrhosis, and 74.0% accurate and 41.1% comprehensive on questions on hepatocellular carcinoma.^31^ In ophthalmology, 2 clinicians assessed chatbot responses to questions on vernal keratoconjunctivitis, finding potentially dangerous responses related to treatment, such as omitting information on key adverse effects.^32^ However, it is likely that safer responses could have been produced using careful prompt design. For example, our prompt to the chatbot sought to eliminate false confidence through explicit instruction to “refer the patient to see their ophthalmologist” if “not highly confident in their response to the patient.” To our knowledge, only 1 prior study directly compared advice produced by an LLM chatbot with advice written by humans. Ayers et al^14^ collected data from an online social media forum, Reddit’s AskDocs subreddit, where users ask questions to moderator-verified health care professionals. One hundred ninety-five user questions and responses from an LLM chatbot and physicians were reviewed in a side-by-side fashion by an expert panel, who preferred chatbot over physician responses for 78.6% of questions. They also rated chatbot responses of higher quality and empathy.^14^ A strength of our study is that we also were able to compare physician and chatbot answers to patient questions; in addition, we masked our expert reviewers as to the origin of the answer and found that neither chatbot nor human-written responses were superior.

The implications of this study are significant for the field of ophthalmology and health care more broadly. With the increasing use of digital technologies in health care, including chatbots and other AI-powered tools, it is crucial to assess the accuracy, safety, and acceptability of these systems to both patients and physicians. Regardless of whether such tools are officially endorsed by health care providers, patients are likely to turn to these chatbots for medical advice, as they already search for medical advice online.^12^ While LLM-based systems are not designed to replace human ophthalmologists, there may be a future in which they augment ophthalmologists’ work and provide support for patient education under appropriate supervision. For example, LLMs could engage with patients prior to their ophthalmologist appointment, offering preliminary information on common eye health concerns. After the visit, LLMs could summarize the key points, allowing the ophthalmologist to direct their attention toward the more complex and nuanced aspects of the patient’s needs. This approach could empower the patient with personalized education while saving the ophthalmologist valuable time that could be applied to more complex and challenging cases. Additionally, LLM integration could enhance clinical workflows by augmenting electronic health record communication systems, such as In Basket (Epic Healthcare Systems).^33^ LLMs can draft messages for patients asking for medical advice in an asynchronous fashion, and can generate suggested responses for common queries or requests from patients or other care team members, a feature particularly useful for routine inquiries or administrative tasks. LLMs could also potentially improve access to health care advice for patients who may not have access to an ophthalmologist, particularly in underserved areas; indeed, some patient questions explicitly mentioned that they turned to the Eye Care forum for advice because they did not have easy access to a local ophthalmologist. However, prior to deployment in clinical settings, these potential use cases would need rigorous study in specific contexts to determine their feasibility and safety.

While LLMs undoubtedly hold tremendous promise, it is crucial to examine its potential drawbacks and the potential harm. LLMs are prone to generating incorrect text, known as “hallucinations.”^34^ In our study, an example that may be considered a hallucination could be the chatbot’s assertion that “removal of the cataract can cause a decrease in the size of the eye,” made in response to a patient query about whether cataract surgery could “shrink” the eye (eTable 3 in Supplement 1). Although this study demonstrates that the overall potential harm of chatbot-generated ophthalmology advice does not significantly differ from human-written advice, it still shows that LLM chatbots can generate outputs with the potential to cause harm. This potential harm is compounded by how the output generated by chatbots often exhibits a striking resemblance to human-written text; although our results suggested that raters were more likely to rate human answers as coming from humans and AI answers as coming from AI, the overall accuracy was still fairly low and for some individual ophthalmologists was less than 50%. Hence, LLM output would be even more likely to deceive nonexpert patients into believing that that content was written by humans, leading to a false sense of trust. Thus, the ideal approach for clinical applications of LLMs may be in aiding ophthalmologists, rather than serving as a patient-facing AI that substitutes their judgment. Moreover, although the chatbot was instructed to answer as a human and not to reveal its identity as an AI for the sake of the current study, any advice provided by LLMs in actual deployment should disclose its AI-generated nature. Another potential harm revolves around patient data, protected health information (PHI). To generate advice that is tailored to the patient’s unique circumstances, PHI would need to be input to the LLM. As ChatGPT is not an open model, it is important to note that OpenAI’s privacy policy explains that they “may collect Personal Information that is included in the input.”^35^ In the clinical deployment of LLMs, policies should include strategies to protect PHI.

### Limitations

While this study provides important insights into the performance of LLM chatbots in providing ophthalmic advice, there are several limitations that must be acknowledged. The study evaluated a sample of questions from a single online forum, answered by a small population of 9 volunteer ophthalmologists, and it is unclear how representative these questions and their answers are of the broader population of eye care questions that patients may have. The human ophthalmologists were volunteering their time to answer these anonymous questions on the medical forum, and likely these answers would not be representative of the typical doctor-patient interaction that occurs when there is an established relationship. In addition, neither the responding physician nor the AI has any medical context surrounding the question; in an established doctor-patient relationship, the doctor can take into account the entire medical context of the patient to answer their questions, whereas it is not clear whether chatbots have such capabilities of ingesting large amounts of medical context for each patient. Therefore, it remains uncertain how LLMs may perform in settings outside of the Eye Care forum. Moreover, the chatbot was specifically prompted to be empathetic; different prompting strategies may produce different types of results. In addition, the study focused on the accuracy and safety of chatbot-generated advice, but did not evaluate patient satisfaction or other factors that may influence the uptake and use of AI-powered health care tools. The study relied on the assessment of a small panel of ophthalmologists, and it is possible that other clinicians may have different perspectives on the quality and usefulness of LLM-generated advice. Additionally, although GPT-3.5—the LLM underlying the chatbot in this study—was trained on a large corpus of publicly available text from books, websites, and articles, the exact details regarding the specific data sets used for training GPT-3.5 are not publicly disclosed. It is thus possible that the model’s training set included data from the Eye Care forum itself. Finally, ophthalmology is a field heavily driven by eye examination and imaging; future studies may evaluate the quality of answers generated when inputs include images of the eye submitted by the patient. This will soon be possible with the latest iteration of LLM chatbots based on GPT-4, which accepts both text and image prompts.^36^

## Conclusions

LLMs hold significant promise for improving the quality and efficiency of patient care with its ability to generate responses to often complex and nuanced medical queries, as demonstrated in this study of patients’ ophthalmology queries. Additional research is needed to assess patient attitudes toward LLM-augmented ophthalmology, to evaluate clarity and acceptability of LLM-generated answers from the patient perspective, test the performance of LLMs in a greater variety of clinical contexts, and to determine an optimal manner of utilizing LLMs that is ethical and minimizes harm. Furthermore, it may soon be possible to develop domain-specialized LLMs via fine-tuning models on multimodal ophthalmologic data. Ultimately, the undeniable reality is that LLMs have emerged and are accessible to the general public. We intend for this study to catalyze more extensive and nuanced dialogue and joint efforts surrounding the use of LLMs in ophthalmology among various health care stakeholders, including patients, clinicians, researchers, and policy makers. The primary goal is to prudently leverage these early research findings to shape the responsible implementation of LLMs in the field of ophthalmology.

## Notes

Supplement 1. **eTable 1.** Prompt Used to Prime ChatGPT to Answer Ophthalmology Questions **eTable 2.** Questionnaire for Ophthalmologist Reviewers **eTable 3.** Examples of Problematic AI Answers **eTable 4.** Examples of Problematic Human Answers

Supplement 2.
Data Sharing Statement
