# Supplementary material for: Comparison of Ophthalmologist and Large Language Model Chatbot Responses to Online Patient Eye Care Questions
Source: JAMA Netw Open. 2023 Aug 22;6(8):e2330320. doi: 10.1001/jamanetworkopen.2023.30320 (PMC10445188; doi:10.1001/jamanetworkopen.2023.30320)
Supplement: Supplement 2. — Data Sharing Statement [file jamanetwopen-e2330320-s002.pdf]

Data Sharing Statement

Bernstein. Comparison of Ophthalmologist and Large Language Model Chatbot Responses to Online Patient Eye Care Questions. *JAMA Netw Open*. Published August 22, 2023.
doi:10.1001/jamanetworkopen.2023.30320

Data
Data available: Yes
Data types: Data (not involving human participants)
How to access data: Data is already publicly available on this forum:
https://www.medhelp.org/forums/Eye-Care/show/90
When available: With publication

Supporting Documents
Document types: None

Additional Information
Who can access the data: Anyone may access this publicly available data.
Types of analyses: Publicly available data available for any analyses
Mechanisms of data availability: It is already publicly available.
Any additional restrictions: None
